# A highly reproducible quantitative viral outgrowth assay for the measurement of the replication-competent latent HIV-1 reservoir

**DOI:** 10.1038/srep43231

**Published:** 2017-02-24

**Authors:** Axel Fun, Hoi Ping Mok, Mark R. Wills, Andrew M. Lever

**Affiliations:** 1 Department of Medicine, University of Cambridge, Cambridge, UK

## Abstract

Cure of Human Immunodeficiency Virus (HIV) infection remains elusive due to the persistence of HIV in a latent reservoir. Strategies to eradicate latent infection can only be evaluated with robust, sensitive and specific assays to quantitate reactivatable latent virus. We have taken the standard peripheral blood mononuclear cell (PBMC) based viral outgrowth methodology and from it created a logistically simpler and more highly reproducible assay to quantify replication-competent latent HIV in resting CD4^+^ T cells, both increasing accuracy and decreasing cost and labour. Purification of resting CD4^+^ T cells from whole PBMC is expedited and achieved in 3 hours, less than half the time of conventional protocols. Our indicator cell line, SupT1-CCR5 cells (a clonal cell line expressing CD4, CXCR4 and CCR5) provides a readily available standardised readout. Reproducibility compares favourably to other published assays but with reduced cost, labour and assay heterogeneity without compromising sensitivity.

Over the last two decades advances in antiretroviral therapy (ART) have transformed infection with Human Immunodeficiency Virus (HIV) from a lethal disease into a manageable chronic condition for the majority of patients with access to high-quality treatment^1^. Despite this success, patients must adhere to life-long therapy since cessation of treatment inevitably results in rebound of plasma viraemia and restitution of disease progression^2^,^3^,^4^,^5^. The predominant source of recrudescent virus is reactivation from a stable reservoir of latently HIV infected resting CD4^+^ T cells which is unaffected by ART and as such prevents eradication of HIV^6^,^7^. Current efforts to cure HIV infection or to achieve therapy-free remission aim at depleting or, preferably, eradicating this latent population^8^.

Accurate quantitation of the latent viral load is critical for the evaluation of these cure strategies. Whilst the bulk of resting CD4^+^ T cells reside in tissues, the latent HIV reservoir is usually measured in peripheral blood resting CD4^+^ T cells for reasons of accessibility. In these long-lived cells HIV persists as integrated proviruses giving the latent HIV population an estimated half-life of 44 months^9^. A variety of techniques are used to quantitate latent HIV including PCR based assays for total HIV and integrated proviral DNA, ultrasensitive single-copy RNA assays, inducible multiply-spliced HIV RNA and culture-based viral outgrowth assays^10^,^11^,^12^,^13^,^14^. Most proviruses are defective and there is poor correlation between these assays^15^. There is no agreement as to which assay approximates best to a biologically meaningful measure of the latent viral load^10^. However, the consensus opinion is that the quantitative viral outgrowth assay, which determines the size of the replication-competent, inducible proviral reservoir in resting CD4^+^ T cells, represents a definitive minimal estimate of its ‘true’ size and is clinically relevant for recrudescence and disease progression^15^,^16^.

The standard quantitative viral outgrowth assay measures replication-competent latent HIV by co-cultivation of *ex vivo* activated resting CD4^+^ cells with PBMCs from HIV negative donors^13^. Although this is a powerful methodology, it has some major drawbacks. The assay is laborious, time consuming and expensive. Heterogeneity of expression of CCR5 on cells from different seronegative donors affects the sensitivity of the assay. These factors combine to make the standard viral outgrowth assay unwieldy for studies with large sample numbers. In clinical trials that compare samples at multiple time-points, as occurs in many of the current eradication studies, assay reproducibility is essential^17^.

We report a streamlined viral outgrowth assay that uses a dual co-receptor expressing cell line, SupT1-CCR5, to replace the PBMC co-culture and employs a single-step resting CD4^+^ T cell purification from peripheral blood with a custom antibody cocktail. These modifications significantly reduce labour and cost and improve assay stability. Our quantitative viral outgrowth assay is easy to perform, robust, relatively inexpensive and can be used for small studies in labs with limited experience with outgrowth assays, or for large scale studies, without the need for extensive human resources.

## Results

### Rapid purification of resting CD4^+^ T cells from whole blood

The highly purified resting CD4^+^ T cells required for the viral outgrowth assay are conventionally obtained in three steps: 1) isolation of PBMCs from whole blood using density gradient centrifugation, 2) negative selection from PBMCs to enrich for total CD4^+^ T cells using a commercially available antibody cocktail followed by 3) depletion of activated CD4^+^ T cells, commonly by targeting cell-surface activation markers CD25, CD69 and HLA-DR. Latently infected cells are rare thus typically large blood volumes are required for the viral outgrowth assay and isolation of resting CD4^+^ T cells generally requires 6–8 hours.

To expedite this process we used SepMate-50 tubes for the isolation of PBMCs from whole blood. They permit easier layering of the blood:PBS mixture onto the density medium and allow for shorter centrifugation times at higher speeds with use of the centrifuge brakes, cutting processing time by as much as 2 hours. The two-step procedure for purifying resting CD4^+^ T cells from PBMCs is widely used because there are as yet no commercially available kits for the selection of resting CD4^+^ T cells. Negative selection kits for total CD4^+^ T cells are available from several manufacturers and depletion of activated cells can be achieved in various ways such as direct removal using anti-CD25/CD69/HLA-DR coated beads or staining for these markers and subsequent depletion using beads targeting the conjugated fluorophore. We depleted activated CD4^+^ T cells using FITC conjugated antibodies against these three markers and removed them with anti-FITC magnetic beads. Alternatively, we isolated highly purified resting CD4^+^ T cells using a custom antibody cocktail which consisted of a CD4^+^ T cell isolation kit supplemented with anti-CD25/CD69/HLA-DR.

The efficacy of both purification methods was tested on PBMCs that were isolated from an apheresis cone and stimulated for 3 days with 10 U/ml Interleukin-2 (IL-2) and 1 μg/ml Phytohaemagglutinin - Leucoagglutinin (PHA-L). This resulted in expression of CD25 and CD69 on >50% of CD4^+^ T cells and HLA-DR on >3% of CD4^+^ T cells, levels far higher than usually observed in clinical samples (ranges in our patient cohort: CD25 0.4–16.8%; CD69 0.2–1.5%; HLA-DR 0.5–11%). Equal amounts of activated PBMCs were then processed using either the two-step or one-step protocol. Even under these unphysiological conditions, both methods resulted in highly purified resting CD4^+^ T cells (Supplementary Figure 1).

**Figure 1:**
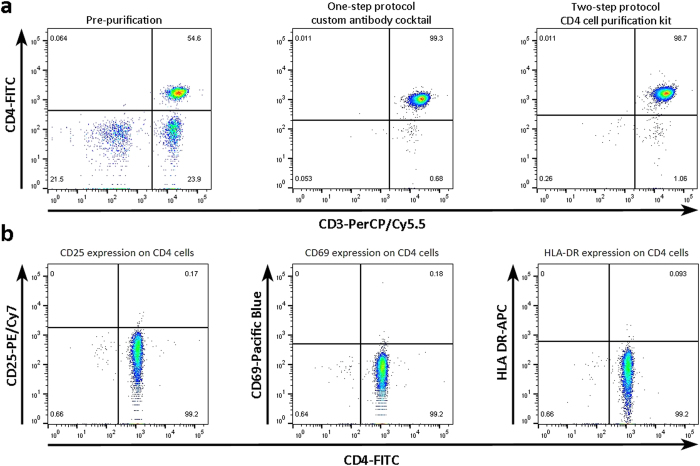
The custom antibody kit yields highly purified resting CD4^+^ T cells. (**a**) Resting CD4^+^ T cells obtained with the one-step protocol (middle panel) or total CD4^+^ T cells with the two-step protocol (right panel) were stained with anti-CD3-PerCP/Cy5.5 and anti-CD4-FITC and their purity was analysed by flow cytometry. Both methods yielded equally pure CD4^+^ T cell populations. (**b**) To test if activated CD4^+^ T cells were efficiently depleted by the custom antibody kit, isolated cells were stained with anti-CD4-FITC, anti-CD25-PE/Cy7, anti-CD69-Pacific Blue and anti-HLA-DR-APC and their purity was analysed by flow cytometry. No activated cell contamination was observed.

When tested on clinical samples, both procedures yielded equally pure populations of resting CD4^+^ T cells (Fig. 1) with similar recovery rates while the one-step protocol reduced processing time from roughly 2 hours to 45 minutes. The conventional two-step procedure resulted in an average purity (±standard deviation) of 97.8% (±1.6%) resting CD4^+^ T cells (n = 20, range 93.5–99.7%) and the one-step procedure using the custom antibody kit returned an average purity of 98.5% (±1.1%) resting CD4^+^ T cells (n = 14, range 96.1–99.5%). There was no statically significant difference between the two purification methods (p = 0.12, Mann-Whitney test). With this new method, highly purified resting CD4^+^ T cells could be obtained from whole blood in no more than 3 hours.

### Virus released from infected CD4^+^ T cells replicates more efficiently in SupT1-CCR5 cells compared to CD8-depleted seronegative donor PBMCs

We compared the ability of SupT1-CCR5 cells with that of CD8-depleted PBMCs from seronegative donors to amplify virus released from infected primary CD4^+^ cells by co-culturing each with serial dilutions of infected CD4^+^ T cells. SupT1-CCR5 cells are SupT1 cells which endogenously express CD4 and CXCR4 and were engineered to stably express CCR5. Primary CD4^+^ T cells were isolated from a single donor and infected with either laboratory strain LAI (X4-tropic virus), BaL (R5-tropic) or clinical isolate MCV (R5-tropic). After 21 days of co-culture viral replication was assayed in each individual well by p24 ELISA.

**Figure 2:**
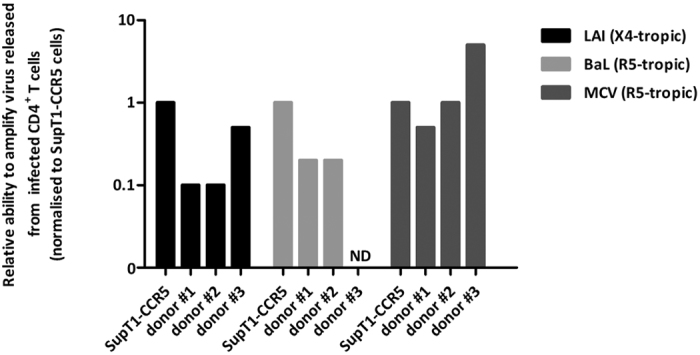
Ability of different amplifier cells to support viral replication of virus released from infected cells. Serial dilutions of infected CD4^+^ T cells (supplemented to a total of 500000 CD4^+^ T cells with uninfected CD4^+^ T cells) were co-cultured with 5 × 10^5^ SupT1-CCR5 cells or 1.34 × 10^6^ CD8-depleted PBMCs from three unselected healthy donors. After 21 days of co-culture mimicking assay conditions, viral production was measured by HIV p24 ELISA. Results were normalised to the lowest number of infected cells that sustained viral replication in SupT1-CCR5 cells. A reading higher than 1 indicates the donor PBMCs were able to amplify virus from fewer infected cells than the SupT1-CCR5 cells and a reading lower than 1 that they required more infected input cells than SupT1-CCR5 cells. (ND - not detected). No viral replication was detected for donor 3 at the highest concentration of BaL infected cells.

SupT1-CCR5 cells were able to amplify LAI virus released from 10 fold fewer infected cells than were donor #1 and #2 CD8-depleted PBMCs and half that of donor #3 CD8-depleted PBMCs (Fig. 2). They also amplified virus released from BaL infected cells more efficiently and required 5 fold fewer infected cells than donor #1 and #2 PBMCs. Emphasising the heterogeneity of PBMCs as an amplifying cell population, the CD8-depleted PBMCs from donor #3 were not able to amplify virus from BaL infected cells in this experiment. In contrast, these cells were superior in amplifying virus from MCV infected cells, requiring 5 fold fewer infected cells to become p24 positive than SupT1-CCR5 and donor #2 cells. CD8-depleted PBMCs from donor #1 required twice the number of infected cells to become positive compared to SupT1-CCR5 and donor #1 cells and 10 times more than donor #3 CD8-depleted PBMCs.

The inconsistent replication efficiency of the two different R5-tropic viruses seen with donor #3 CD8-depleted PBMCs demonstrates the capricious ability of donor derived PBMCs to support viral replication when using unselected seronegative donors. By contrast, SupT1-CCR5 cells were excellent amplifier cells, better able to support viral replication and performed more uniformly than CD8-depleted PBMCs from unmatched, unscreened healthy donors.

### SupT1-CCR5 cells are as efficient as CD8-depleted seronegative donor PBMCs at amplifying reactivated virus in the viral outgrowth assay

A drawback of the conventional PBMC based co-culture assay is the heterogeneity of CCR5 expression on donor PBMCs. Using PBMCs from healthy donors that exhibit high levels of CCR5 expression after mitogen stimulation can greatly enhance the sensitivity of the PBMC co-culture based outgrowth assay^18^. Therefore it is common practice to screen for donors with high levels of CCR5 expression or even use matched patient-donor pairs in the standard PBMC co-culture assay^17^. While this may improve the sensitivity of the assay it also increases time, labour, complexity and cost of each assay. Using individual matched donors also prevents inter assay benchmarking between different donor-patient pairs. Replacing the CD8-depleted healthy donor PBMCs that have to be added to the assay on days 2, 9 and 16 with a cell line stably expressing CD4, CXCR4 and CCR5 that only requires a single addition on day 2 could increase sensitivity, inter-assay homogeneity and substantially reduce labour and cost of each assay.

**Figure 3:**
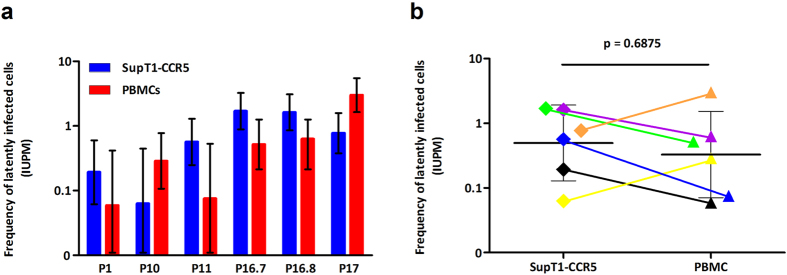
Direct comparison of the SupT1-CCR5 cell based assay with the ‘standard’ PBMC based assay. (**a**) Purified resting CD4^+^ T cells were split in half and parallel assays run with SupT1-CCR5 cells (blue bars) and CD8-depleted PBMCs as amplifier cells (red bars). PBMCs were from unselected seronegative donors. Patient identifiers are depicted on the X-axis, P16.7 and P16.8 were samples from the same patient taken at different time points. Error bars indicate the 95% confidence interval (CI) for each individual assay. (**b**) There was no statistically significant difference in IUPM values between the two types of amplifier cells. Colours indicate the corresponding samples from the same patient and are connected by a coloured line. Horizontal bars represent the geometric mean of each data set and the error bars indicate the 95% CI. P-value was calculated using a Wilcoxon matched pairs test.

To evaluate the SupT1-CCR5 based viral outgrowth assay, resting CD4^+^ T cells were isolated from virologically suppressed HIV-1 positive individuals and divided equally for parallel outgrowth assays using either SupT1-CCR5 cells or CD8-depleted healthy donor PBMCs. Six samples from five patients (one patient was assayed twice) were used for direct comparisons (Fig. 3). The healthy donor PBMCs for each assay were from six different donors obtained from the NHS Blood and Transplant Centre. In all samples replication competent virus was detected using both SupT1-CCR5 cells and healthy donor PBMCs. In 4/6 samples SupT1-CCR5 cells gave a higher frequency of latently infected cells, reported as infectious units per million cells (IUPM) and in 2/6 the PBMC co-cultures resulted in a higher IUPM (Fig. 3a). There was no statistically significant difference in IUPM between the SupT1-CCR5 cell viral outgrowth assay and the standard PBMC co-culture assay (Fig. 3b).

### The SupT1-CCR5 based viral outgrowth assay shows a high level of reproducibility

**Figure 4:**
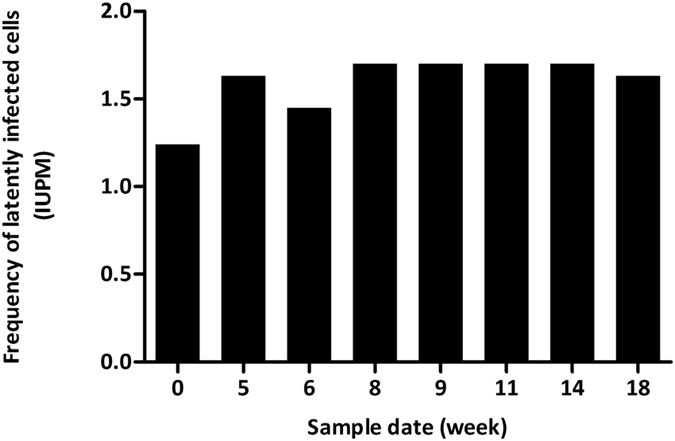
The SupT1-CCR5 cell based viral outgrowth assay has excellent reproducibility. Blood was collected from a patient undergoing regular venesection for the treatment of haemochromatosis (P16, Table 1). SupT1-CCR5 cell-based viral outgrowth assays were performed on a set of 8 blood samples from this patient to assess the reproducibility of the assay. The date of venesection is depicted on the X-axis with the first sample set as week 0.

To evaluate the reproducibility of the SupT1-CCR5 based viral outgrowth assay we determined the IUPM in two HIV positive individuals at multiple time-points. Both patients were undergoing regular venesection for the coincidental treatment of haemochromatosis. We tested 8 samples over 4 months from one patient (P16, Table 1). This patient was virologically suppressed and no HIV RNA blips were reported over this period. In addition, we tested 3 samples taken over 19 months from a second patient (P6, Table 1). The patient was virologically suppressed throughout this period. All 8 samples from P16 had an IUPM between 1.240 and 1.701 indicating very high assay reproducibility with a standard deviation of only 0.17 (95% confidence interval (CI) 0.11–0.34) (Fig. 4). The 3 samples from P6 which were taken at months 0, 3 and 19 were consistently negative suggesting a very small inducible replication-competent latent reservoir size of <0.052 IUPM which was at the lower limit of detection for the assay with an input of 1.92 × 10^7^ resting CD4^+^ T cells^19^.

### Measurement of the replication-competent latent reservoir in a heterogeneous patient cohort by the SupT1-CCR5 based viral outgrowth assay

To evaluate the performance of the assay in a clinical setting 25 patients were recruited. Of these patients 20 were stably suppressed and 5 were viraemic. The baseline characteristics of these patients are listed in Table 1.

**Figure 5:**
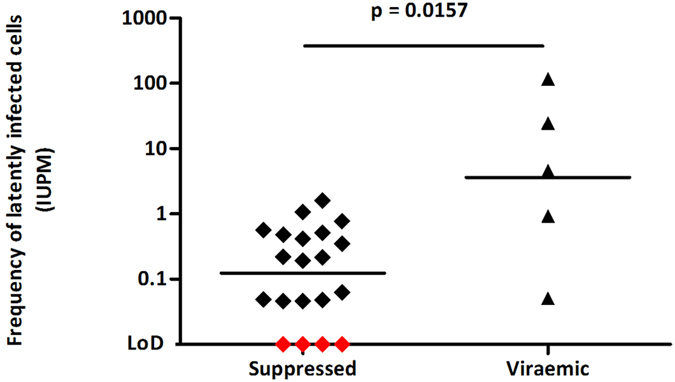
Size of the replication-competent latent HIV reservoir as determined by the SupT1-CCR5 cell based viral outgrowth assay. The assay was tested on a cohort of 25 patients, 20 of whom were virologically suppressed and 5 untreated patients (Table 1). Viraemic patients had a statistically significant larger latent HIV reservoir than aviraemic patients. Horizontal bars represent the geometric mean of each data set. LoD indicates the limit of detection. The samples indicated in red were negative and therefore have an IUPM < 0.046–0.052, the LoD of the assay with the number of input cells used in these four assays. P-value was calculated using a Mann-Whitney test.

The heterogeneity of the cohort and variations in the amount of blood drawn resulted in a broad range of resting CD4^+^ T cell recovery with an average of 3.59 × 10^7^ cells obtained (range 1.2 × 10^7^–10^8^). Replication-competent virus was detected in 21/25 patients with a median latent reservoir size of 0.417 IUPM. Consistent with published data, viraemic patients had a larger reactivatable HIV population than virologically suppressed patients (Fig. 5)^14^,^20^,^21^. The size of the latent reservoir (log transformed IUPM) in suppressed patients inversely correlated with time of virological suppression (<400 copies/ml) (n = 12, Spearman r = −0.8947, p < 0.001), concordant with the current understanding of early establishment and slow decay of the latent HIV pool^20^,^22^,^23^,^24^,^25^,^26^. There was also a clear trend supporting an inverse correlation between the size of the latent reservoir (log_10_ IUPM) and time on treatment (n = 16, Spearman r = −0.4676, p = 0.0678) and virological suppression <50 copies/ml (n = 12, Spearman r = −0.3193, p = 0.3117). Although these did not reach statistical significance, this was probably due to the small data set and the virological suppression (<50 copies/ml) data was possibly confounded by viral blips.

**Figure 6:**
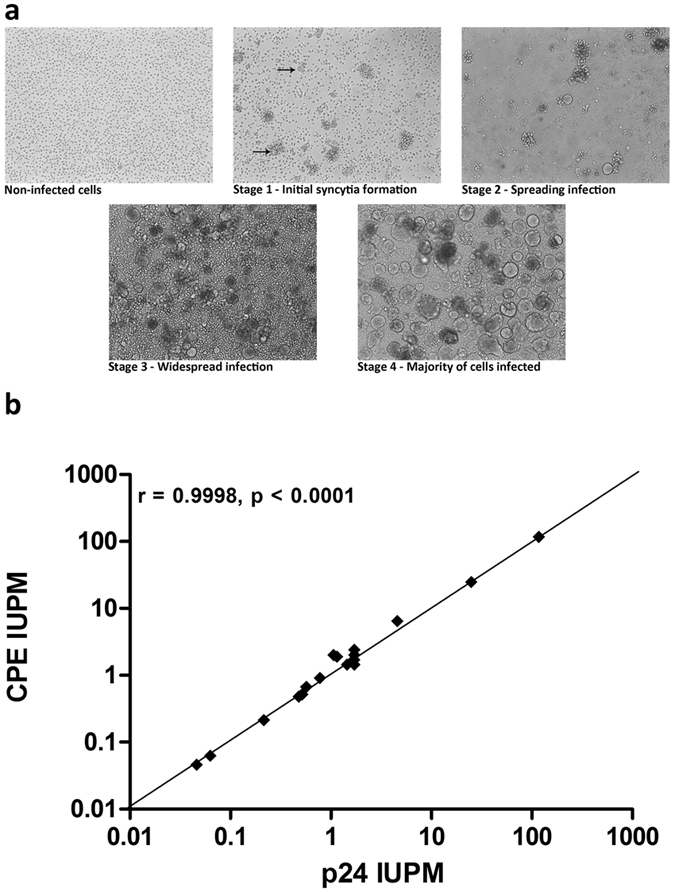
SupT1-CCR5 cells induce syncytia formation upon HIV infection. (**a**) During culture, SupT1-CCR5 cells cluster together in clumps. Mixing the cells disrupts the clumps and creates a single cell suspension which can aid the detection of syncytia. The different stages of progressive infection from non-infected cells to widespread CPE in a SupT1-CCR5 culture infected with HIV from reactivated patient-derived CD4^+^ T cells. The black arrows in the Stage 1 panel point the first appearances of syncytia in the culture. (**b**) Correlation between p24 and CPE read-out. From 18/25 assays comprehensive CPE data was available. The number of positive wells scored by observation of syncytia was converted into an IUPM for each assay and compared to the IUPM based on p24 production. There was a strong correlation between the two read-out methods (Pearson’s correlation coefficient, r = 0.9998).

SupT1-CCR5 cells readily form syncytia during HIV-1 infection (Fig. 6a). We speculated whether the cytopathic effect (CPE) could be used as an alternative read-out to the p24 ELISA. Detailed observation was made of CPE from 18/25 assays and an IUPM was calculated for each based on the number of positive wells scored by this method. CPE results showed a near exact correlation with the p24 results (Pearson’s correlation coefficient, r = 0.9998) (Fig. 6b). From several CPE positive wells viral RNA was isolated and sequenced and all viruses tested had an R5-tropic genotype (http://coreceptor.geno2pheno.org/) indicating that the SupT1-CCR5 cells readily sustain infection with R5-tropic virus and form syncytia in a similar manner to infection with X4-tropic virus. All viruses tested to date induce syncytia formation in these cells. The wide variety of viruses tested include laboratory reference viruses LAI, NL_4-3_ (X4-tropic), BaL (R5-tropic), HIV-2 (SVR clone, induces smaller syncytia than the HIV-1 strains but was clearly discernible) and >30 clinical isolates. In the 18 assays with comprehensive CPE data available, the start of syncytia formation was generally observed around the earliest time p24 was measurable in a well, suggesting that both methods have similar sensitivities. Thus visual scoring of CPE could be used as a reliable corroborative or alternative read-out for all viral isolates.

## Discussion

Developments in HIV latency research and latency reversal have led to a renewed interest in clinical studies aimed at virus eradication and have dramatically increased the use and importance of accurate quantitative viral outgrowth assays. The current standard assay requires leukapheresis or a very large blood draw to obtain sufficient resting CD4^+^ T cells. CD8-depleted HIV negative donor PBMCs must be prepared at least twice, either freshly isolated, or thawed and subsequently stimulated, to maximise permissiveness for HIV infection at least 2 days prior to the day they are needed. The cost of a single assay can therefore amount to several thousands of dollars in consumables alone. Expression levels of CCR5 on HIV negative donor cells vary significantly between individuals and it has been shown that using donors that express high levels of CCR5 greatly enhances the sensitivity of the assay^18^. This has led to screening for donors with high levels of CCR5 expression or using matched patient-donor pairs exhibiting optimal viral replication. This involves extensive pre-screening adding further to the complexity and cost of the assay. Matched patient-donor pairing also inhibits standardisation between assays and laboratories. Such factors make the assay prohibitive for laboratories with limited resources or for use in large studies with a high sample number.

We have updated and streamlined the quantitative outgrowth assay to reduce cost and labour while maintaining sensitivity and improving reproducibility. A single individual can perform and maintain multiple assays simultaneously. By using specialised tubes for density gradient PBMC isolation and replacing the two-step resting CD4^+^ T cell isolation by a single-step procedure, the processing time from whole blood to highly purified resting CD4^+^ T cells is reduced from 6–8 to only 3 hours. Substituting the seronegative donor PBMC co-culture with a cell line based co-culture that only requires a single addition of cells on day 2 significantly decreases labour and cost and increases manageability. Read-out for positive wells is flexible and can be adjusted to the user’s requirements. We opted for p24 ELISA as it is simple, robust and cheap. PCR-based techniques can be a more sensitive alternative and may allow for a shorter co-culture time^14^. The system also offers an alternative option to further reduce labour and cost by visual observation of syncytia formation. We show that CPE data correlate closely with the p24 read-out.

Assay reproducibility is paramount in the clinical trial setting. After statistical analysis of 160 standard assays using matched patient-donor pairs on samples from 37 patients, Crooks *et al*. reported the standard deviation of log transformed latent viral load (log_10_ IUPM) to be 0.38 and calculated a >6-fold decrease as a reliable parameter to identify an effect of the intervention^17^. Expressed as log_10_ IUPM, our unique reproducibility data on multiple samples at short intervals from the same patient shows a standard deviation of only 0.049 (95% CI 0.032–0.100), suggesting our assay is capable of detecting even smaller changes in the replication-competent latent HIV reservoir.

Alternative published approaches have also sought to simplify and shorten the gold standard viral outgrowth assay. One measures the frequency of cells with inducible multiply-spliced HIV RNA (TILDA)^11^ and another uses a viral outgrowth assay that replaces the required seronegative donor PBMCs with a cell line (MOLT-4/CCR5)^14^. While TILDA is very rapid (2 days) and requires less than a million input cells, it detects cells that have inducible multiply-spliced HIV RNA but cannot distinguish if these cells produce the more biologically relevant replication-competent virus. It also samples the total CD4^+^ T cell pool, a very different population than the resting CD4^+^ T cells used in quantitative viral outgrowth assays, exemplified by a median frequency of infected cells 48 times higher than measured by a quantitative viral outgrowth assay^11^. The MOLT-4/CCR5 based viral outgrowth assay uses an RT-PCR targeted at polyadenylated HIV RNA to detect virus production^14^. While PCR is more sensitive than a p24 ELISA, it is more complex and expensive and in contrast to SupT1-CCR5 cells, the MOLT-4/CCR5 cells express CCR5 under the control of the G418 selectable marker. G418 inhibits polypeptide synthesis and the effect on patient-derived CD4^+^ T cells and virus production from reactivated cells is not known, although it is not reported to influence the assay. As the MOLT-4/CCR5 cells are also described as highly susceptible to HIV^14^ they are a plausible alternative reporter line for the SupT1-CCR5 cells. Similar to other versions of the quantitative outgrowth assay, our assay represents a minimal estimate of the size of the inducible replication-competent latent HIV reservoir. Not all latently infected cells are induced to produce virus upon a single round of stimulation. Negative wells that received an additional round of PHA stimulation occasionally became positive whereas their paired counterparts that did not receive additional stimulation remained negative (data not shown), similar as described by Ho *et al*.^16^.

Our quantitative viral outgrowth assay is simple, manageable, highly reproducible and cost-effective. Processing time to obtain highly purified resting CD4^+^ T cells from whole blood is reduced compared to other viral outgrowth assays and is achievable within 3 hours. It requires a single addition of amplifier cells on day 2 and no selective medium. Including the p24 ELISA, the cost of a single assay is <£600 (GBP) and can be substantially lower if scaled up for multiple assays. The manageability and relatively low assay cost make it more accessible for limited resource settings or for larger scale studies requiring a large number of samples to be processed.

## Materials and Methods

### Patient cohort and blood draws

All participants gave written informed consent and this study was approved by the National Health Services (NHS) Health Research Authority (UK) under REC reference 12/SC/0679. All experimental procedures were approved by the institutional review board of the University of Cambridge and were performed in accordance with the relevant guidelines.

Twenty-five patients were included in the study of whom 20 were aviraemic and 5 had detectable plasma HIV-1 RNA (Table 1). The average age of the cohort was 52.2 (median 51, range 37–73) years and consisted of 17 (68%) men and 8 (32%) women. From the majority of patients (20/25) blood was drawn using standard needles and syringes and transferred to 50 ml tubes containing 5 ml 100 U/ml heparin as anticoagulant (volume collected: 125–200 ml, median 170 ml). From 5/25 blood was collected using a blood collection bag containing citrate phosphate dextrose adenine solution (CPDA-1) as anticoagulant (volume collected: 300–600 ml). Both collection and preservation methods were compatible with the downstream applications.

### Cell culture

SupT1-CCR5 cells and PBMCs were maintained in RPMI 1640 with L-glutamine (supplemented with 10% Foetal Calf Serum (FCS). SupT1-CCR5 cells were split 1:10 twice a week and media was refreshed in PBMCs cultures according to the pH indicator of the medium and depended on cell density.

### PBMC isolation

PBMCs were isolated from whole blood by density gradient centrifugation using Lymphoprep (Alere Technologies AS, Oslo, Norway). Anticoagulated blood was diluted 1:1 with PBS and mixed thoroughly. The blood:PBS mixture was layered (up to 34 ml/tube) on top of 15 ml Lymphoprep in a Sepmate-50 tube (StemCell Technologies, Grenoble, France). Tubes were centrifuged for 10 minutes at 1200 g, with the brake on. The lymphocyte containing layer was decanted in new 50 ml tubes and centrifuged for 5 minutes at 900 g to pellet the cells. Cells were resuspended and washed twice with PBS.

### Purification of resting CD4^+^ T cells

PBMCs were resuspended in Recommended Medium (RM; PBS + 2% FCS + 1 mM EDTA).

#### Two-step protocol

CD4^+^ T cells were purified by negative selection using the EasySep Human CD4+ T Cell Enrichment Kit (StemCell Technologies) following the manufacturer’s protocol. Resting CD4^+^ T cells were obtained by staining activated cells using anti-human CD25-FITC, CD69-FITC and HLA-DR-FITC and subsequent depletion using the EasySep Human FITC Positive Selection Kit (StemCell Technologies). Purity of the obtained resting CD4^+^ T cells was assessed by flow cytometry using antibodies specific for CD3-PerCp/Cy5, CD4-FITC, CD8-Pacific Blue, CD25-Pe/Cy7, CD69-Pacific Blue and HLA-DR-APC.

#### One-step purification using a custom antibody cocktail

Resting CD4^+^ T cells were purified using a custom antibody cocktail (StemCell Technologies) which consisted of the EasySep Human CD4+ T Cell Enrichment Kit supplemented with anti-CD25/CD69/HLA-DR. A change was made to the manufacturer’s protocol to increase recovery. Briefly, cells were resuspended at a concentration of 5 × 10^7 ^cells/ml in RM in a 14 ml 17 × 100 mm tube and custom antibody cocktail was added at 50 μl/ml, mixed and incubated for 15 minutes at room temperature. RapidSpheres were added at 75 μl/ml, mixed and incubated for 5 minutes at room temperature. The cell suspension was brought to a total volume of 5 ml (for <2 × 10^8^ cells) or 10 ml (for >2 × 10^8^ cells) with RM and mix by pipetting up and down and placed in the magnet (‘The Big Easy’ EasySep magnet, StemCell Technologies) for 5 minutes. The unbound fraction was decanted into a new tube and the RapidSpheres were resuspended in 5 ml RM and placed in the magnet again for 5 minutes and decanted in the same tube. The second fraction occasionally contained magnetic bead contamination and therefore the combined fraction was placed in the magnet again for 5 minutes and decanted in a new tube to obtain highly purified resting CD4^+^ T cells. PBMC isolation and analysis by flow cytometry were identical to the two-step protocol.

### Limiting dilution assay

Purified resting CD4^+^ T cells were cultured in 10 ml RPMI medium containing 10% FCS supplemented with 20 nM efavirenz and 100 nM raltegravir for 2 to 4 days to allow for the degradation of unintegrated viral DNA. On ‘day 1’ of the assay, cells were washed thoroughly to remove any traces of antiretrovirals and subsequently 5-fold dilutions of resting CD4^+^ T cells were prepared and stimulated with a 10-fold excess of ɣ-irradiated (5000 rad) allogeneic PBMCs and 2 μg/ml PHA-L and plated in the following manner: 6 wells with 2.5 × 10^6^ resting CD4^+^ T cells/well in a 6 well plate, 12 wells with 0.5 × 10^6^ resting CD4^+^ T cells/well in a 12 well plate and 12 wells with 0.1 × 10^6^ resting CD4^+^ T cells/well in another 12 well plate. Throughout the assay cells were maintained in RPMI medium containing 10% FCS supplemented with 10 U/ml IL-2. On day 2, 24 hours after stimulation all supernatant was carefully aspirated from the cells to remove the PHA and incubated for 2 hours in RPMI medium containing FCS and IL-2. After 2 hours the supernatant was aspirated again and SupT1-CCR5 cells were added in fresh media. 6 well plates received 6 ml media containing a total of 1 × 10^6^ SupT1-CCR5 cells per well and 12 well plates received 3 ml media containing a total of 0.5 × 10^6^ SupT1-CCR5 cells per well. Half of the media was refreshed on days 9, 12, 16 and 19 and experiments were terminated on day 23. Supernatants were harvested at several time-points and analysed for HIV-1 p24 production by ELISA.

To compare the SupT1-CCR5 co-culture assay with the standard PBMC co-culture assay, CD8-depleted HIV negative donor PBMCs were prepared 48–72 hours before adding the target cells on day 2. In short, apheresis cones were obtained from the NHS Blood and Transplant Centre and leukocytes were obtained by density-gradient centrifugation. CD8^+^ T cells were depleted using CD8 Microbeads (Miltenyi, Bisley, UK). A portion of CD8-depleted PBMCs was subsequently viably frozen and the remainder was stimulated with 10 U/ml IL-2 and 2 μg/ml PHA-L for 48–72 hours. On day 2 of the assay they were washed to remove all mitogens and added to the serial dilutions of stimulated CD4^+^ T cells: 6 well plates received 4 × 10^6^ CD8-depleted PBMCs per well and 12 well plates received 1.34 × 10^6^ CD8-depleted PBMCs per well in 6 and 3 ml media respectively. The frozen CD8-depleted PBMCs were thawed in two batches on days 7 and 14 and stimulated as described before and added to the cultures in a similar fashion on days 9 and 16.

### Comparing viral replication efficiency in SupT1-CCR5 cells with HIV negative donor PBMCs

Laboratory strain virus stocks (LAI and BaL) were prepared by transfecting 293 T cells with the relevant plasmids and harvesting cell-free supernatant after 48 hours. MCV was a clinical HIV strain isolated from patient P16 and cultured in SupT1-CCR5 cells to create a high titre virus stock. The created virus stock appeared to be clonal in the V3 region as determined by Sanger sequencing and had a CCR5 co-receptor usage as predicted by the Geno2pheno algorithm (http://coreceptor.geno2pheno.org/) with a false positive rate (FPR) of 3.8% (cut-off 2.5%). CCR5 co-receptor usage was confirmed by replication experiments in U87.CD4.CXCR4 and U87.CD4.CCR5 cells (data not shown). Viral titres were determined by adding serial dilutions of virus to TZM-bl cells and measuring luciferase activity after 48 hours.

PBMCs from HIV negative donors were obtained as described above. 2 × 10^8^ PBMCs where infected with an equivalent infectious dose with either lab strain LAI (X4-tropic virus), BaL (R5-tropic) or clinical isolate MCV (R5-tropic) and cultured for 4 days in the presence of 1 μg/ml PHA-L and 10 U/ml IL-2. In addition to the infected PBMCs, 5 × 10^8^ uninfected PBMCs from the same donor were cultured in similar conditions. After 4 days the total CD4^+^ T cells were purified from the cultures using the EasySep Human CD4+ T Cell Enrichment Kit. Serial dilutions of infected cells were made in a 12 well plate consisting of 500000, 100000, 50000, 10000, 5000, 1000, 500, 100, 50, 10, 5 or 1 infected cells per well. Uninfected CD4^+^ T cells were added to a total of 500000 CD4^+^ T cells in all wells. To mimic assay conditions, these cells were then stimulated with a 10-fold excess of ɣ-irradiated allogeneic PBMCs and 2 μg/ml PHA-L. After 24 hours cells were washed and the amplifier cells were added, either 0.5 × 10^6^ SupT1-CCR5 cells or 1.34 × 10^6^ PHA-stimulated CD8-depleted donor PBMCs per well. CD8-depleted PBMCs from three different unselected seronegative donors were tested as amplifier cells. Co-cultures were maintained for 21 days. In the PBMC co-cultures, stimulated CD8-depleted PBMCs (from the same donor) were added on days 2, 9 and 16 as opposed to a single addition of SupT1-CCR5 cells on day 2, similar to the assay conditions. Supernatants were harvested at various time-points to analyse HIV-1 p24 production by ELISA.

## Additional Information

**How to cite this article**: Fun, A. *et al*. A highly reproducible quantitative viral outgrowth assay for the measurement of the replication-competent latent HIV-1 reservoir. *Sci. Rep.* **7**, 43231; doi: 10.1038/srep43231 (2017).

**Publisher's note:** Springer Nature remains neutral with regard to jurisdictional claims in published maps and institutional affiliations.

## Supplementary Material

Supplementary Figure 1

## Figures and Tables

**Table 1:** Patient characteristics.

Pt. ID	Age (years)	Sex	Time since diagnosis (years)	Nadir CD4 count (cells/mm^3^)	CD4 count at sample time (cells/mm^3^)	VL at sample time (c/ml)	Time on ARV (months)	Time VL < 50 c/ml (months)	Time VL < 400 c/ml (months)	Therapy regimen (current)	Recovered resting CD4 cells (×10^6^)	IUPM
P1	42	F	10	N/A	450	<50	114	N/A	N/A	N/A	42	0.193
P2	48	M	23	N/A	350	<50	234	N/A	N/A	N/A	65	0.350
P3	48	F	9	N/A	530	<50	102	N/A	N/A	N/A	65	0.220
P4	63	M	7	300	820	<50	63	57	73	TDF, FTC, DRV/r	22	neg
P5	51	M	27	N/A	980	<50	270	>123	>123	TDF, FTC, RAL, DRV/r	50	0.049
P6	66	M	12	140	470*	<50	143	143	143	ddI, 3TC, EFV	19*	neg
P7	52	F	16	110	420	<50	180	171	171	ABC, 3TC, NVP	40	0.048
P8	57	M	4	270	580	<50	55	55	56	TDF, FTC, ATV/r	22	neg
P9	53	M	20	360	640	<50	92	42	167	TDF, FTC, DRV/r	29	0.046
P10	73	M	9	370	460	<50	92	49	111	TDF, FTC, DRV/r	39	0.063
P11	50	F	20	N/A	740	<50	242	>78	>78	d4T, ddI, DRV/r	34	0.567
P12	57	M	10	9	370	<50	124	124	124	TDF, FTC, EFV	17	0.215
P13	54	F	14	170	>1500	<50	167	42	173	TDF, FTC, EFV	100	0.046
P14	58	M	12	280	430	<50	150	>75	>75	ABC, 3TC, EFV	21	neg
P15	62	F	6	160	520	<50	68	24	68	TDF, FTC, NVP	22	1.062
P16	61	M	8	30	440*	<50	91	28	70	TDF, FTC, ATV/r	27*	1.594*
P17	55	M	6	320	650	<50	70	65	75	TDF, FTC, EFV	66	0.775
P18	46	M	11	220	850	<50	133	59	86	ddI, 3TC, ATV/r, RAL	80	0.417
P19	48	F	11	N/A	1070	<50	28	N/A	N/A	TDF, FTC, EFV	32	0.516
P20	47	M	4	90	320	<50	42	5	41	TDF, FTC, ATV/r	25	0.479
VP1	42	M	11	N/A	480	4,538	none	viraemic	viraemic	Therapy naïve	20	0.933
VP2	37	M	3	410	570	76,193	none	viraemic	viraemic	Off therapy	20	0.051
VP3	40	M	4	N/A	390	134,535	none	viraemic	viraemic	Therapy naïve	12	117
VP4	45	F	6	N/A	370	6,892	none	viraemic	viraemic	Off therapy	12	24.848
VP5	51	M	3	230	N/A	8,000	none	viraemic	viraemic	Off therapy	16	4.574

P - suppressed patient; VP - viraemic patient; N/A - data not available; ARV - antiretroviral therapy; VL – viral load; c/ml – copies/ml; IUPM - infectious units per million cells; neg - negative result (no replication-competent HIV detected). ^*^Average of multiple samples from a single patient. Antiretroviral drugs: TDF - tenofovir disoproxil fumarate; FTC - emtricitabine; DRV/r - darunavir boosted with ritonavir; RAL - raltegravir; ddI - didanosine; 3TC - lamivudine; EFV - efavirenz; ABC - abacavir; NVP - nevirapine; ATV/r - atazanavir boosted with ritonavir; d4T - stavudine.
