# Supplementary material for: A highly reproducible quantitative viral outgrowth assay for the measurement of the replication-competent latent HIV-1 reservoir
Source: Sci Rep. 2017 Feb 24;7:43231. doi: 10.1038/srep43231 (PMC5324126; doi:10.1038/srep43231)
Supplement: Supplementary Figure 1 [file srep43231-s1.pdf]

Supplementary Information

A highly reproducible quantitative viral outgrowth assay for the measurement of the replication-competent latent HIV-1 reservoir

Axel Fun[1], Hoi Ping Mok[1], Mark R Wills[1*] and Andrew M Lever[1*]

[1]Department of Medicine, University of Cambridge, Cambridge, UK

Supplementary Figure 1

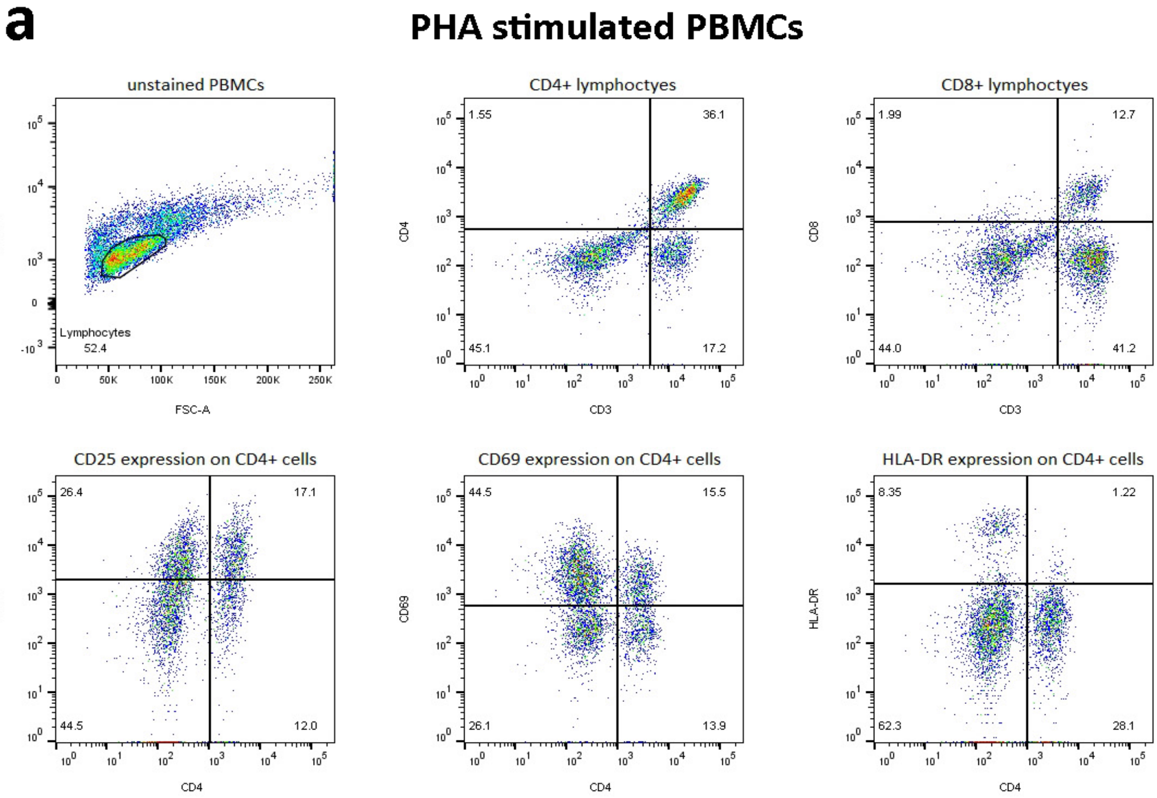

b Purified total CD4+ T cells

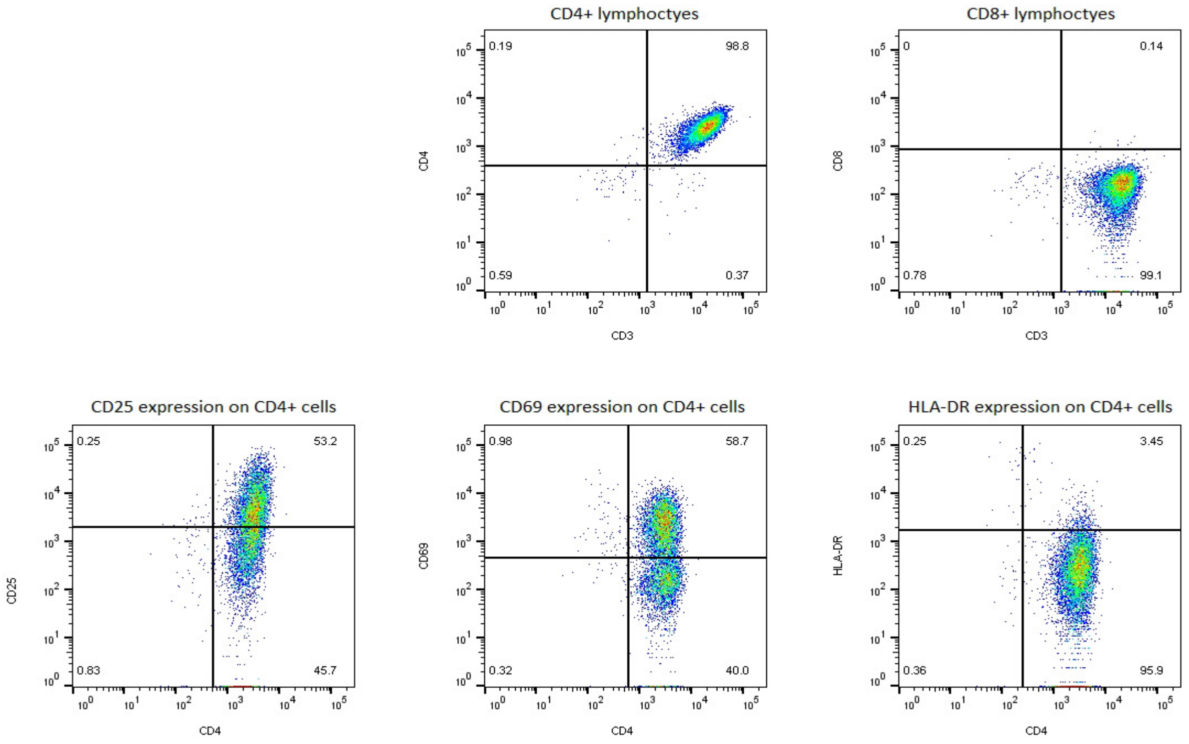

c Purified resting CD4+ T cells after activated cell depletion

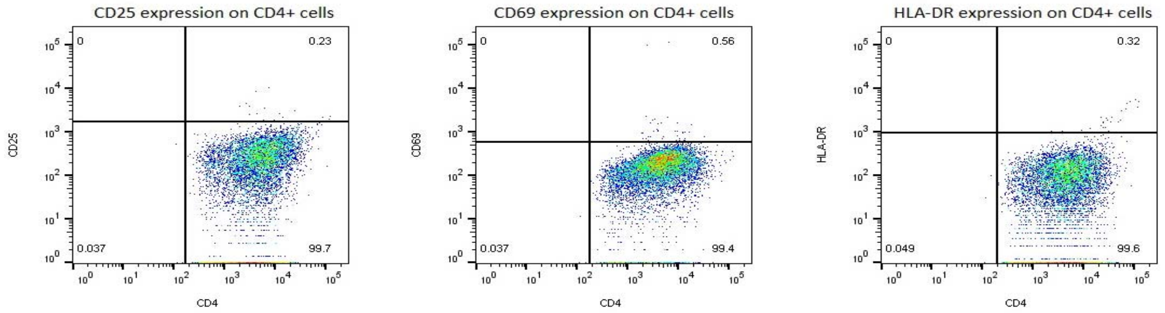

d

Resting CD4+ T cells purified in a single step

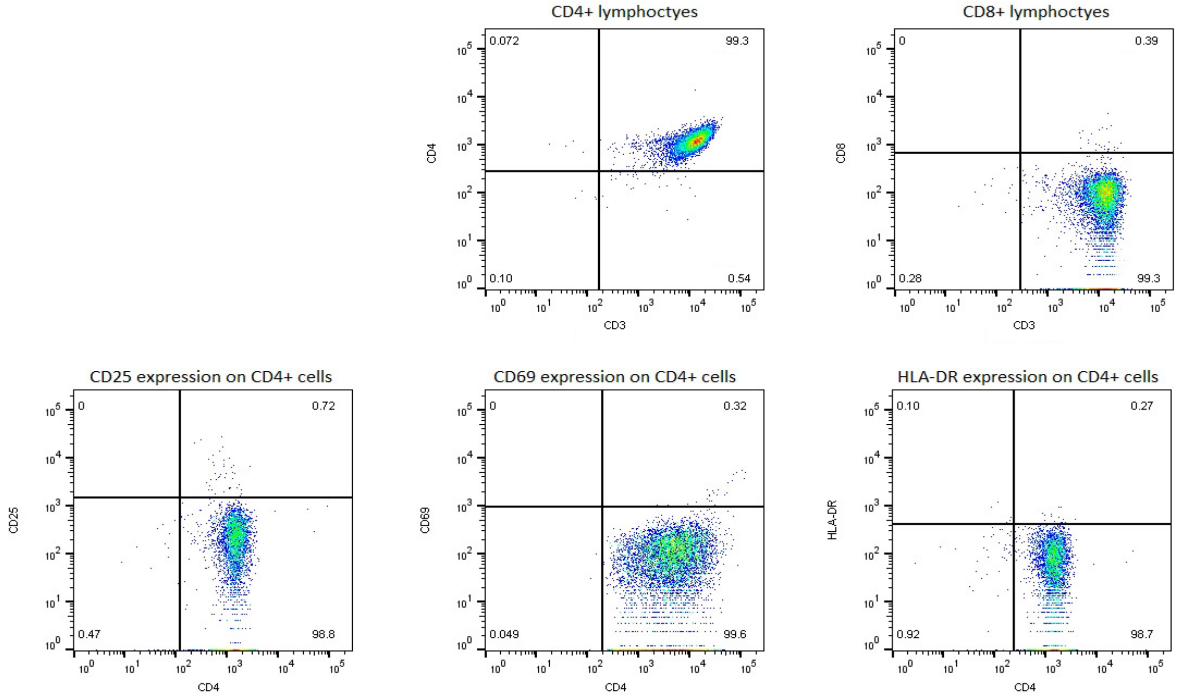

Supplementary Figure 1. Purification of resting CD4$^+$ T cells from highly activated PBMCs with the one-step custom antibody kit and the standard two-step procedure. PBMCs were stimulated for 3 days with 10 U/ml IL-2 and 1 µg/ml PHA-L. Cells were stained with either anti-CD3-PerCP/Cy5.5, anti-CD4-FITC and anti-CD8-PAcific Blue or anti-CD4-FITC, anti-CD25-PE/Cy7, anti-CD69-Pacific Blue and anti-HLA-DR-APC to analyse their purity by flow cytometry. (**a**) Mitogen stimulation resulted in high expression levels of CD25 and CD69 and moderate expression of HLA-DR on lymphocytes. (**b**) Total CD4$^+$ T cells were isolated with a commercial isolation kit yielding highly purified CD4$^+$ T cells and FACS analysis revealed an expression of CD25 and CD69 on >50% of CD4$^+$ T cells and HLA-DR on >3% of CD4$^+$ T cells. (**c**) Activated cells were depleted with FITC conjugated antibodies against activation markers CD25, CD69 and HLA-DR using anti-FITC magnetic beads. This resulted in highly purified resting CD4$^+$ T cells. (**d**) Resting CD4$^+$ T cells were obtained in a single step using a custom antibody cocktail with a yield and purity identical to the standard two-step procedure.
